# Dyslipidemias: Prevalence and Associated Factors among Lactating Women in a Lower- and Middle-Income Country, Ghana

**DOI:** 10.1155/2023/6280494

**Published:** 2023-11-18

**Authors:** Gideon Kofi Helegbe, Saeed Jabactey Abdullah, Baba Sulemana Mohammed

**Affiliations:** ^1^Department of Biochemistry and Molecular Medicine, School of Medicine, University for Development Studies, Ghana; ^2^Department of Nutritional Sciences, School of Allied Health Sciences, University for Development Studies, Ghana; ^3^Department of Pharmacology and Toxicology, School of Pharmacy and Pharmaceutical Sciences, University for Development Studies, Ghana

## Abstract

**Background:**

Dyslipidemia, an abnormally high level of lipids in the blood, has a negative impact on the health status of the individual and has lately emerged as a major public health concern, especially for low- and middle-income countries (LMIC) globally, including Ghana. However, it is still unclear what the burden and drivers of these lipid abnormalities are, especially among lactating women in the Upper West of Ghana. Thus, this study is aimed at determining the prevalence of dyslipidemia and its associated factors among lactating mothers in the Wa Municipality of Ghana. *Methodology*. A cross-sectional study was conducted from May to June 2020 in 8 health facilities within the Wa Municipality. Multistage and simple random sampling methods were used to select the facilities and the 200 study subjects. Sociodemographic data were collected using questionnaires, while blood samples were taken to determine the lipid profile of participants. Dietary patterns were also assessed using the Food Frequency Questionnaire (FFQ). Data were processed and analyzed using SPSS 17 software (SPSS, Inc., Chicago, IL). The chi-square test and multiple regression analysis were performed to determine the predictors associated with the various types of dyslipidemia, with statistical significance set at a *p* value < 0.05.

**Results:**

The prevalence of hypercholesterolemia (LDL-C), hypo-HDL-cholesterolemia, and hypertriglyceridemia (TG) was 57%, 59%, and 22%, respectively. Chi-square and multinomial regression analysis revealed that duration of lactation (*X*^2^ = 3.95, *p* = 0.047), religion (AOR = 0.375, 95% CI 0.144–0.978, *p* = 0.045), low income (AOR = 0.116, 95% CI 0.026–0.514, *p* = 0.005), middle income (AOR = 0.163, 95% CI 0.044–0.600, *p* = 0.006), and alcohol intake (AOR = 6.312, 95% CI 1.108–35.949, *p* = 0.038) were associated with LDL-C, while age (AOR = 0.963, 95% CI 0.910-1.019, *p* < 0.001) and educational status (AOR = 0.365, 95% CI 0.140–0.954, *p* = 0.040) predicted HDL status.

**Conclusion:**

Dyslipidemia is common among lactating mothers of Wa Municipality, and it is predicted by lifestyle factors. Furthermore, future research to look at a larger sample size on dyslipidemia during lactation is recommended.

## 1. Introduction

Dyslipidemia is the abnormal value of any of the transportable lipid forms, namely, high-density lipoprotein (HDL), low-density lipoprotein (LDL), very low-density lipoprotein (VLDL), and triglyceride (TG), in the bloodstream [1, 2] or as a combination of abnormalities of lipoprotein concentration in the bloodstream [3]. Dyslipidemia is of more public health burden, especially for high- and middle-income countries [4], due to its association with atherosclerosis [5] and the attendant coronary heart disease (CHD) [6] and strokes.

Dyslipidemia is categorized into primary and secondary dyslipidemia, where the primary condition refers to the overproduction, a lack of production of or excessive clearance of HDL, or the inability of the body system to clear some lipoproteins arising from genetic defects during the lipoprotein metabolism process [7], with familial hypercholesterolemia, hypertriglyceridemia, and hypocholesterolemia as examples [8]. Secondary dyslipidemia is related to the acquired form of the disorder and is associated with factors such as physical inactivity and consumption of saturated fatty diets, hypothyroidism, diabetes mellitus, obstructive liver disease, hypertension, thiazide diuretics, and progestin [9]. Drinking coffee, advanced age, raised fasting blood sugar, and vegetable intake have also been reported as significant predictors of dyslipidemia among a population of women using contraceptives in eastern Ethiopia [10].

The prevalence of dyslipidemia varies in different localities ranging from 66.7% to 89.0% [5, 11, 12], and in Ghana, its public health importance has been described with data from independent studies [13, 14]. A study also revealed that dyslipidemia is more prevalent among the female population relative to the male population of Turkey [15].

Pregnancy and menopause are also known to affect the blood lipid profiles of women [11]. During pregnancy, the metabolic activity is physiologically adjusted to accommodate increased progesterone and estrogen serum concentrations, hence leading to an increase in the metabolism of lipids [16]. Meanwhile, breastfeeding improves the lipid profile, as indicated in some studies, thereby having the potential to reduce the risk of cardiovascular diseases [17, 18]. This suggests breastfeeding to be an important factor in overcoming the attendant weight gain and associated problems such as hypertension, among others.

Lactation is important immediately following childbirth and is characterized by an elevation in progesterone and estrogen serum concentrations. The interaction of these hormones alters the physiological and hormonal balance of the mother, thus compromising their health status [19]. While evaluation of dyslipidemia has been done on the general population, few studies exist to evaluate lipid abnormalities among lactating mothers, and little to no data is recorded on lactating mothers in northern Ghana. This study therefore sought to determine the prevalence of dyslipidemias and associated factors among lactating mothers in the Wa Municipality of northern Ghana, a lower middle-income country.

## 2. Methodology

### 2.1. Study Area

**Figure 1:**
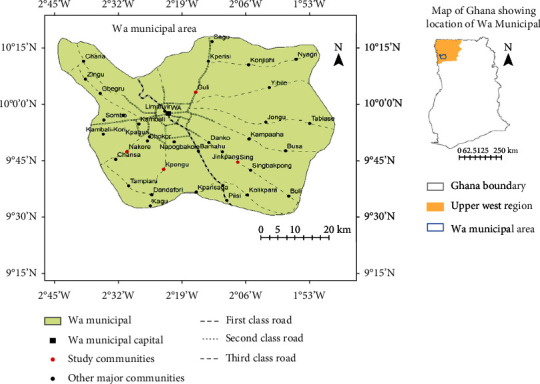
Map of Wa Municipality (Google images; date accessed August 2019).

The study was carried out in the Wa Municipality of the Upper West Region (UWR), located in the northwestern part of Ghana (Figure 1). This study area was chosen for the fact that the data on this dyslipidemia among lactating mothers in the study area was scanty. The region has four boundaries, with Burkina Faso to its north and la Cote d'Ivoire to the western part of the region, while the newly created Savannah Region is with the southern boundary, with the Upper East Region on the eastern part of the UWR.

### 2.2. Study Design, Target Population, and Sampling

**Figure 2:**
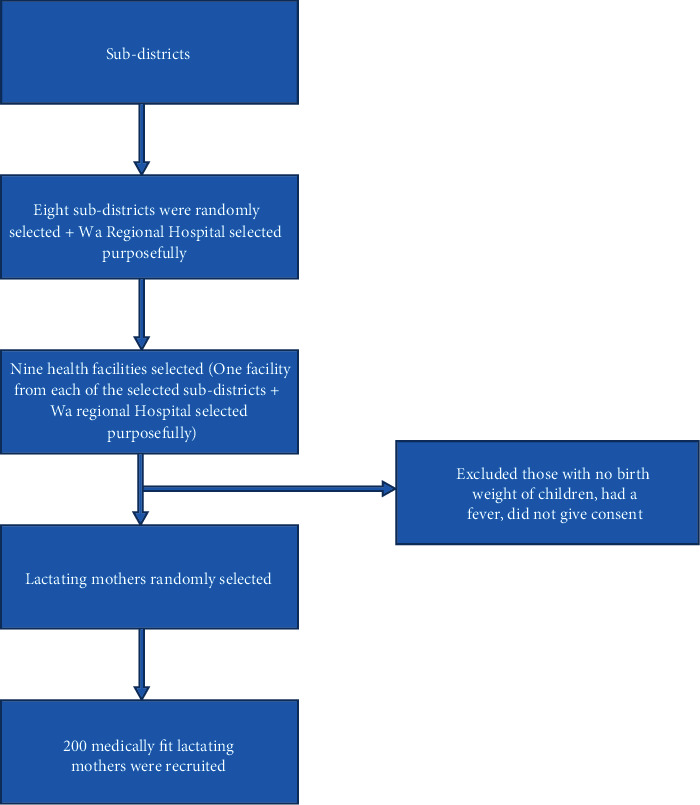
Flow diagram of the study procedure. Medically fit are those without fever or no medical problem for which they will have to be referred to the medical doctor for treatment.

A cross-sectional design was adopted to address the objective of the study. The lactating women within Wa Municipality constituted the targeted population of the study, which was estimated to be 4,950 based on the number of annual deliveries in the municipality [20]. A multistage sampling was used in selecting eight subdistricts from the ten subdistricts in the Wa Municipality, and these subdistricts were Bamahu, Charia, Dobile, Kambali, Kperisi, Kpongu, Wa South, and Wa North. From each of these selected subdistricts, one facility that provided child welfare clinic (CWC) services was chosen. For the purpose of this study, a total of nine health facilities were selected, including eight chosen randomly: Bamahu Hospital, Adolescent Clinic, Market Clinic, Dobile South CHPS, Gbegru CHPS, Kpaguri CHPS, Konjiehi CHPS, and Dandafuro CHPS. The Wa Regional Hospital was included in this selection as it serves as a key referral hospital. Two hundred (200) medically fit, consenting lactating mothers who visited any of the nine selected health facilities for CWC services during the time of the study were recruited.  Figure 2 gives the flow of the study and subject recruitment.

### 2.3. Sample Size Determination

The sample size required to be representative of the population was determined using Cochran's formula for sample size determination [21]

(1) $$\begin{array}{c} n=\frac{Z^{2}\text{PQ}}{d^{2}}, \end{array}$$

where *n* represents the sample size. At a confidence interval of 95%, hence, *Z* is equivalent to 1.96. *P* represents the estimated proportion, which in this case is 93.4% [11]. *Q* is equal to 1, *P* is equal to 1, and 0.0934 = 0.9066. *d* is the margin of error, and this case was chosen as 5% since the confidence interval is 95% = 0.05.

(2) $$\begin{array}{c} n=\frac{1.96^{2}\times 0.934\times 0.9066}{0.05^{2}},\\ n=\frac{1.96^{2}\times 0.934\times 0.066}{0.05^{2}},\\ n=\frac{3.8416*0.061644}{0.0025},\\ n=\frac{0.2368}{0.0025},\\ n=94.7. \end{array}$$

This is approximated to be 95.0.

But since the source population is less than 10,000, in estimating the sample size:

(3) $$\begin{array}{c} Nf=\frac{n}{1}+\frac{n}{N}, \end{array}$$

where *n* is 95 and *N* is 10,000.

(4) $$\begin{array}{c} Nf=\frac{95}{1}+\frac{95}{10,000}. \end{array}$$

Therefore, the minimum sample size required for the study was estimated at 95. However, a 10% upward adjustment was made to the estimated minimum sample size to accommodate an assumed 10% nonresponse rate, giving the final sample size of 104.

To ensure a fair distribution of the number of study subjects recruited, the number of participants was weighted, and the final figure was computed as indicated in Table 1.

### 2.4. Inclusion and Exclusion Criteria

Only lactating women who had visited the CWC during the period of the study were medically fit, had medically fit children, and consented were included in the study. Study subjects were excluded from the study if they had no record of the birth weight of their children, had a fever, and did not consent to be part of the study.

### 2.5. Data Collection

Data was collected from April to May 2020, and the biochemical analysis of the blood samples was conducted in June 2020. A one-day training was given to data enumerators on how to administer the questionnaire, ensure the privacy of the participants, and ensure the confidentiality of their information. A pretested questionnaire was administered to the study participants to elicit data on sociodemographic characteristics and lifestyle characteristics, together with dietary assessments of the respondents using the Food Frequency Questionnaire (FFQ) [22]. To evaluate the lipid profile of the study participants, a minimum of 2 ml of venous blood samples was taken from each study participant and given a unique code. The venous blood samples were centrifuged at 3,500 rpm for ten minutes, and the collected serum was stored at -20°C until it was analyzed. On the day of analysis, serum samples were removed from the freezer to thaw, and serum lipid concentration was quantified using the AX-200 Automatic Biochemistry Analyzer.

### 2.6. Data Processing and Analysis

Microsoft Excel (2013) was used to process and clean the data that was generated, which was exported into SPSS version 17 software (SPSS, Inc., Chicago, IL, USA) for statistical analysis. The number of lactating women with elevated plasma concentrations of LDL and TG (triglycerides) and lowered plasma concentration levels of HDL were reported as proportions. The sociodemographic characteristics of the respondents such as age, marital status, and type of residence were analyzed by employing descriptive statistics. The prevalence of dyslipidemia for the independent variables (covariates) was presented as frequencies and percentages. Continuous variables were summarized using means, with standard deviations or medians, and ranges, where appropriate. Pearson's chi-square was used to test for any associations between variables. Multiple logistic regression was used to test for the predictive effects of independent variables on the dependent variable, dyslipidemia, once other covariates were controlled. A statistical significance of *p* < 0.05 was set for all the statistical analyses, and all odds ratios were presented with 95% confidence intervals.

### 2.7. Ethical Considerations

Ethical clearance was obtained from the Committee on Human Research Publication and Ethics (CHRPE) of the Kwame Nkrumah University of Science and Technology (KNUST), Kumasi, Ghana, with ID number CHRPE/AP/178/22. An introductory letter was obtained from the Department of Nutritional Sciences, University for Development Studies (UDS), and submitted to the Wa Municipal Health Directorate for approval to conduct the study in the selected facilities. Permission was given by the administrators and in-charges of the various facilities that were randomly chosen. Participants in the study were voluntarily and randomly chosen to participate in the study after their informed consent was obtained.

## 3. Results

### 3.1. Background Characteristics of Respondents

In all, 200 lactating women responded to the questionnaires, and their characteristics are summarized in Table 2. The most represented age groups were 28–32 and 33–37 years (56, 28.0%), with those <18 years being the least (1, 0.5%); all respondents were married at the time of the study (200, 100%). The study shows that the majority (150, 75%) of the lactating women were of the Islamic faith, while 172 (86%) had formal education and 28 (14.0%) were noneducated. Furthermore, the majority (156, 78.0%) of the respondents were employed, with the following income status: low income (88, 44.0%), middle income (95, 47.5%), and high income (17, 8.5%). It was also observed that the majority of the study participants (172, 86.0%) had urban residences.

### 3.2. Lifestyle Activities of the Respondents

Table 3 gives an overview of the lifestyle characteristics of lactating mothers. Means of transport and frequency of exercise were evaluated to assess the level of physical activity. It was observed that the majority (137, 68.5%) of the respondents used engine vehicles as their means of transport, with 63 (31.5%) on foot. Almost half of the study participants (93, 46.5%) exercised often (at least 5 times a week), while 60 (30%) had no form of exercise. The majority (183, 91%) of the study participants did not consume alcohol. Most (148, 91.5%) of the women breastfed their children exclusively, which was done for 7 months. One hundred and eighty (180, 90%) of the mothers had an index child with normal birth weight, and 164 (82.0%) of the study participants had normal (i.e., via vaginal) delivery. Furthermore, the majority (184, 92.0%) of the deliveries were singletons.

### 3.3. Sociodemographic Characteristics and Lifestyle Activities Relationship with Lipid Profile of Lactating Women

**Figure 3:**
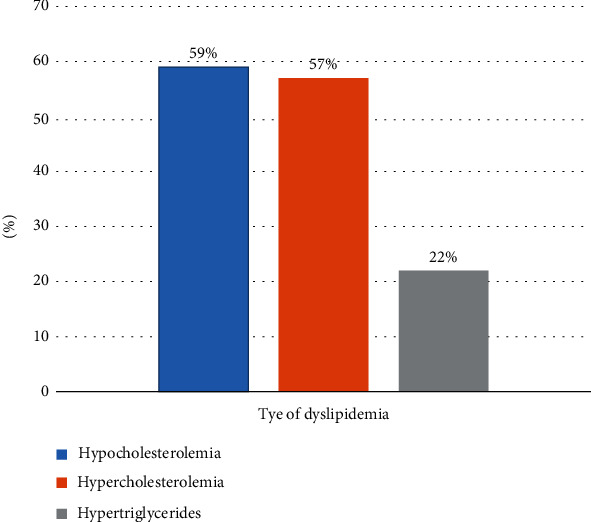
Prevalence of the various types of dyslipidemias.

One hundred and fourteen (114) lactating women had elevated levels of LDL serum cholesterol (LDL-C), suggesting a 57.0% prevalence of hypercholesterolemia, while a 118 (59.0%) prevalence of hypocholesterolemia was also observed due to a low level of high-density lipoprotein cholesterol (HDL-C) (Figure 3). Furthermore, 44 (22.0%) of study participants were observed to have high levels of triglycerides (TG).

The relationship between dyslipidemia and sociodemographic characteristics as well as the lifestyles of respondents are summarized in Table 4. The results indicated that educational status and duration of lactation were associated with dyslipidemia. While educational status was associated with hypocholesterolemia (*X*^2^ = 4.486; *p* = 0.034), duration of lactation was associated with hypercholesterolemia (*X*^2^ = 3.957; *p* = 0.047). A higher proportion of the Muslims were hypercholesterolemic and had elevated LDL (17, 66%). Religion, occupation, residence, distance to work, income status, means of transport, level of exercise, alcohol intake, breastfeeding type, delivery outcome, parity, and delivery type did not show any associations with dyslipidemia.

### 3.4. Factors Associated with Dyslipidemias

Table 5 describes the dietary factors associated with the various dyslipidemias under study. Although the study participants have a wide range of dietary habits, none were significantly associated with dyslipidemia. Meanwhile, by means of multinomial logistic regression analysis (Table 6), it was observed that age (AOR, 0.963, 95% CI 0.910–1.019, *p* < 0.001) and educational status (AOR, 0.365, 95% CI 0.140–0.954; *p* = 0.040) were significantly less likely to be associated with the hypocholesterolemia dyslipidemia (low level of HDL). Low income (AOR, 1.841, 95% CI 0.378–8.955, *p* = 0.450), middle income (AOR, 1.825, 95% CI 0.425–7.849, *p* = 0.418), and no alcohol intake (AOR, 1.568, 95% CI 0.312–7.875, *p* = 0.585) were almost twice likely to be associated with hypocholesterolemia, though not significantly. Religion (AOR, 0.375, 95% CI 0.144–0.978; *p* = 0.045), low income (AOR, 0.116, 95% CI 0.026–0.514; *p* = 0.005), and middle income (AOR, 0.163, 95% CI 0.044–0.600, *p* = 0.006) were significantly less likely to be associated with hypercholesterolemia (elevated LDL), while alcohol intake was six times more likely to be associated with hypercholesterolemia (elevated LDL) (AOR, 6.312, 95% CI 1.108–35.949; *p* = 0.038). While low income (AOR, 2.475, 95% CI 0.291–21.079, *p* = 0.407) and nonexclusive breastfeeding (AOR, 2.019, 95% CI 0.634–5.428, *p* = 0.234) were twice likely to be associated with hypertriglycerides (though not significant), engaging in lactation for >6 months (AOR, 2.704, 95% CI 0.865–8.458, *p* = 0.087) were thrice likely to be associated with hypertriglycerides.

## 4. Discussion

While dyslipidemia in the general population and pregnant women has been documented, little is known about this condition in lactating mothers, especially in the study area, the UWR of Ghana. Even though dyslipidemia was common among the lactating mothers in the study area, it was significantly less associated with certain lifestyle and sociodemographic characteristics.

Dyslipidemia is of great public health concern due to its association with atherosclerotic plaques and consequent CHD and strokes. Thus, the various dyslipidemia types observed in the current study call for a more proactive approach among lactating mothers to reduce mortalities and disabilities linked with lipid metabolism. Elevated levels of LDL-C and hypertriglyceridemia (TG) observed in this study implicate CVD, fat embolisms, and other related ailments of atherosclerosis. LDL that has not been oxidized will not lead to foam cell formation; however, lipid peroxidation of LDL, as it accumulates, makes the LDL a target or ligand for certain receptors, including the scavenger receptor and perhaps a specific receptor for oxidized LDL. These events generate cholesterol-laden foam cells, and oxidized LDL in the cell wall enhances the production of cytokines and growth factors, resulting in monocyte recruitment and the proliferation of smooth muscle cells [24].

The high prevalence (59%) of hypocholesterolemia (HDL-C) in this study gives cause for concern in relation to the physiology of the lactating mother. Higher rates of hypocholesterolemia (HDL-C) were also observed in other studies (60.91%, [25]; 64%, [26]; 72%, [13]; and 61%, [14]). HDL-C functions to scavenge any excess cholesterol within the body; thus, being low is a high risk for cardiovascular disease (CVD) for the participants in this study.

Age was associated with hypocholesterolemia in the current study; however, such was not the case with any of the dyslipidemia types studied in other studies ([27] but varies with [28–30]). Meanwhile, studies have shown that older persons have a higher tendency to develop dyslipidemia [28, 31]. The explanation is that, as one age, the level of physical activity decreases, thus increasing the risk of developing dyslipidemia. Furthermore, the study participants in the current study were relatively younger generation (86% < 37 years).

Enani et al. [32] did not observe any association of religion with dyslipidemia as reported in the current study. Meanwhile, the majority of the study population were Muslims, who are noted to have a strict diet with minimal fat, as noted by the kind of diet reported in this study. Studies have shown that while a higher education level is associated with an increased level of physical activity, low-nutrient and high-calorie food consumption is often noted in women with lower level of education [33]. Such unhealthy practices are high-risk factors for CVD. Thus, the association of education with low-level HDL-C (hypocholesterolemia) in this study and elsewhere [28, 34] is of public health concern. Education provides an opportunity for awareness of safe life habits and independence over one's health decisions, thus boosting self-confidence to have control of one's health style choices.

Individuals with high-income levels were observed to be at higher risk for dyslipidemia [34]. The understanding is that they are accustomed to sedentary lives with less physical activity. It is not clear why hyperlipidemia was associated more with low- and middle-income earners in the current study. It has been established that alcohol intake is associated with increased HDL for those with moderate alcohol intake [35, 36]. However, in other studies, alcohol intake is associated with increased LDL-C [37] and TG [38–40], as observed in the current study. These have implications for public health. Hyperlipidemia is a metabolic defect in diabetics due to the accelerated breakdown of lipid molecules into acetyl-CoA molecules in an attempt to make energy available to the body. Consequently, lipids are resynthesized in the blood from the acetyl-CoA molecules that accumulate in the bloodstream, narrowing the blood vessels and resulting in atherosclerosis and associated CVD. Meanwhile, diabetes was not studied in the current study, which can be explored in further studies.

Physical exercise remains one of the recommendations for those being affected by dyslipidemia. However, no relationship between dyslipidemia and physical exercise was revealed in the current research, while other studies report an association [29, 41]. Although the degree or intensity of exercise was not evaluated, it is important to recommend to lactating mothers that they engage in the types of exercise that will increase metabolism.

The benefits of breastfeeding and its duration in relation to dyslipidemia need to be explored further. This is due to the conflicting opinions being reported regarding the link between the duration of breastfeeding and dyslipidemia. While our study observed dyslipidemia to be associated with a longer duration of breastfeeding as reported elsewhere [28], another study found no time-dependent association between breastfeeding (lactation) and metabolic risk [42]. Meanwhile, central adiposity is known to be associated with high dyslipidemia. This is possible due to the accumulation of visceral fat instead of the subcutaneous fat being linked to hyperlipidemia. Thus, to prevent or reduce lipid metabolism disorders, it is important and necessary to reduce abdominal fat and increase lipid metabolism, hence the need to encourage appropriate exercise for these lactating mothers.

The results of this current study in relation to diet and its association with dyslipidemia were explored, but these were not significant and were in contrast to other studies [32, 43, 44]. The results of those studies also established that the consumption of butter and eggs was associated with hypercholesterolemia, while hypertriglycerides were associated with fish, butter, eggs, and milk consumption. These observations confirm the role of diet in the health of an individual, particularly the development of dyslipidemia, irrespective of the geographical location of the study or individual. It is a known fact that there are some fish types and eggs that will impact the lipid pattern in the body of an individual, either resulting in hyperlipidemia or hypolipidemia. This study, however, did not investigate the food types, which should be explored in further studies. Meanwhile, grains, fish, vegetables, and fruits contain fiber which decreases the absorption of fat and sugar in the small intestine, helping to lower the number of triglycerides in the bloodstream. This should be recommended for lactating women.

Although some limitations were observed in the study, it has several strengths. This is the first of its kind to be conducted in the study area and has provided sound knowledge in the study area. The use of FFQ in accessing their dietary pattern may pose a limitation. Dietary intake is difficult to measure, and any single method cannot assess dietary exposure perfectly. Nutritional biomarkers are valid for objective estimates of dietary exposures in anthropometric and clinical assessments, while the 24HR, DR, dietary history, and FFQ are subjective estimates. Notwithstanding the discussed limitation, FFQs are still widely used as the primary dietary assessment tool in epidemiological studies. Recently, it has been suggested that a combination of methods, such as the FFQ with DRs (or 24HR) or the FFQ with biomarker levels, be used to obtain more accurate estimates of dietary intakes than those of individual methods. Considerable efforts to improve the accuracy and feasibility of large epidemiological studies are still ongoing. In summary, dietary assessment methods should be selected with caution while considering the research objective, hypothesis, design, and available resources [22].

## 5. Conclusions

This study shows that dyslipidemia is prevalent among the lactating mothers of Wa Municipality, which is predicted by some lifestyle factors (age, alcohol intake, income status, religion, and duration of lactation). It is recommended that future studies consider a larger sample size for dyslipidemia during lactation.

## Figures and Tables

**Table 1:** Proportionate distribution of sample size by health facility.

Name of facility	Facility code	Average monthly attendance	Weight (%)	Proportionate sample distribution
Wa Regional Hospital	WRH	308	15.11	30
Bamahu Clinic	BMC	152	7.46	15
Adolescent Clinic	AHC	197	9.67	19
Konjiehi CHPS	KOJC	254	12.46	25
Market Clinic	MARC	388	19.04	38
Dobile south CHPS	DBSC	217	10.65	21
Kpaguri CHPS	KPGC	215	10.55	21
Gbegru CHPS	GGC	102	5.01	10
Dandafuro CHPS	DDFC	205	10.06	21
Total		2,038^∗^	100	200

^∗^Data obtained from the Annual Report for the Upper West Region (Source: Upper West Regional Directorate, 2020).

**Table 2:** Sociodemographic characteristics of respondents (*n* = 200).

Variable	*n* (%)
Age	
<18	1 (0.5)
18-22	14 (7.0)
23-27	45 (22.5)
28-32	56 (28.0)
33-37	56 (28.0)
>37	28 (14.0)
Marital status	
Married	200 (100.0)
Religion	
Christianity	50 (25.0)
Islam	150 (75.0)
Educational status	
Educated	172 (86.0)
Noneducated	28 (14.0)
Occupation	
Employed	156 (78.0)
Unemployed	44 (22.0)
Residence	
Rural	28 (14.0)
Urban	172 (86.0)
Income status	
Low income	88 (44.0)
Middle income	95 (47.5)
High income	17 (8.5)

*n* (%) = number (percentage).

**Table 3:** Lifestyle characteristics (*n* = 200).

Variable	*n* (%)
Means of transport	
Engine vehicle	137 (68.5)
Foot	63 (31.5)
Exercise level	
None	60 (30.0)
Maybe	47 (23.5)
Often	93 (46.5)
Alcohol intake	
No	183 (91.5)
Yes	17 (8.5)
Type of breastfeeding	
Exclusive	148 (74.0)
Nonexclusive	52 (26.0)
Birth weight of index child	
Normal	180 (90.0)
High	20 (10.0)
Parity	
Uniparous	62 (31.0)
Multiparous	138 (69.0)
Delivery outcome	
Singleton	184 (92.0)
Twins	16 (8.0)
Duration of lactation	
6 months and below	51 (25.5)
7 months and above	149 (74.5)
Type of delivery	
Normal	164 (82.0)
Cesarean section	32 (16.0)
Forceps	4 (2.0)

*n* (%) = number (percentage).

**Table 4:** Chi-square analysis of factors associated with the various types of dyslipidemias.

Variable	Hypocholesterolemia *n* (%)		*X* ^2^, *p*	Hypercholesterolemia *n* (%)		*X* ^2^, *p*	Hypertriglyceride *n* (%)		*X* ^2^, *p*
	Yes	No		Yes	No		Yes	No	
Religion									
Christianity	47 (31.3)	103 (68.7)	0.970, 0.325	40 (26.7)	110 (73.3)	0.990, 0.320	18 (12.0)	132 (88.0)	0.613, 0.434
Islam	12 (24)	38 (76)		17 (66.0)	33 (34.0)		4 (8.0)	46 (92.0)	
Educational status									
Educated	46 (26.7)	126 (73.3)	**4.486**, **0.034**	49 (28.5)	123 (71.5)	0.001, 0.993	19 (11.0)	153 (89.0)	0.003, 1.000
Noneducated	13 (46.4)	15 (53.6)		8 (28.6)	20 (71.4)		3 (10.7)	25 (89.3)	
Occupation									
Employed	45 (28.8)	111 (71.2)	0.146, 0.703	44 (28.2)	112 (71.8)	0.030, 0.862	18 (11.5)	138 (88.5)	0.210, 0.789
Unemployed	14 (31.8)	30 (68.2)		13 (29.5)	31 (70.5)		4 (9.1)	40 (90.9)	
Residence									
Rural	9 (32.1)	19 (67.9)	0.109, 0.741	52 (30.2)	120 (69.8)	1.810, 0.179	19 (11.0)	153 (89.0)	0.003, 0.958
Urban	50 (29.1)	122 (70.9)		5 (17.9)	23 (82.1)		3 (10.7)	25 (89.3)	
Distance to work^∗^									
Far	38 (29.2)	92 (70.8)	0.013, 0.909	35 (26.9)	95 (73.1)	0.453, 0.501	11 (8.5)	119 (91.5)	2.445, 0.118
Near	21 (30.0)	49 (70.0)		22 (31.4)	48 (68.6)		11 (15.7)	59 (84.3)	
Income status^∗∗^									
High income	3 (17.6)	14 (82.4)		9 (52.9)	8 (47.1)		2 (11.8)	15 (88.2)	2.569, 0.277
Low income	29 (30.5)	66 (69.5)	1.256, 0.534	25 (26.3)	70 (73.7)	5.447, 0.066	7 (7.4)	88 (92.6)	
Middle income	27 (30.7)	61 (69.3)		23 (26.1)	65 (73.9)		13 (14.8)	75 (85.2)	
Means of transport^#^									
Engine vehicle	43 (31.4)	94 (68.6)	0.745, 0.388	40 (29.2)	97 (70.8)	0.104, 0.747	15 (10.9)	122 (89.1)	0.001, 0.973
Foot	16 (25.4)	47 (74.6)		17 (27.0)	46 (73.0)		7 (11.1)	56 (88.9)	
Exercise level^##^									
Maybe	16 (34.0)	31 (66.0)		24 (25.8)	69 (74.2)		8 (8.6)	85 (91.4)	
None	17 (28.3)	43 (71.7)	0.612, 0.736	18 (30.0)	42 (70.0)	0.666, 0.717	8 (13.3)	52 (86.7)	1.030, 0.598
Often	26 (34.0)	67 (72.0)		15 (31.9)	32 (68.1)		6 (12.8)	41 (87.2)	
Alcohol intake									
No	56 (30.6)	127 (69.4)	1.255, 0.263	54 (29.5)	129 (70.5)	1.074, 0.405	22 (12.0)	161 (88.0)	2.296, 0.226
Yes	3 (17.6)	14 (82.4)		3 (17.6)	14 (82.4)		0 (0.0)	17 (100.0)	
Breastfeeding type									
Exclusive	44 (29.7)	104 (70.3)	0.014, 0.904	41(27.7)	107 (72.3)	0.178, 0.673	15 (10.1)	133 (89.9)	0.435, 0.510
Nonexclusive	15 (28.8)	37 (71.2)		16 (30.8)	36 (69.2)		7 (13.5)	45 (86.5)	
Delivery outcome									
Single	54 (29.3)	130 (70.7)	0.026, 0.873	53 (28.8)	131 (71.2)	0.105, 0.746	21(11.4)	163 (88.6)	0.401, 0.527
Twins	5 (31.3)	11 (68.8)		4 (25.0)	12 (75.0)		1 (6.3)	15 (93.87)	
Parity									
Uniparous	18 (29.0)	44 (71.0)	0.009, 0.923	19 (30.6)	43 (69.4)	0.203, 0.652	8 (12.9)	54 (87.1)	0.332, 0.564
Multiparous	41 (29.7)	97 (70.3)		38 (27.5)	100 (72.5)		14 (10.1)	124 (89.9)	
Type of delivery									
Normal 1	52 (31.7)	112 (68.3)		46 (28.0)	118 (72.0)		17 (10.4)	147 (89.6)	
Cesarean section 2	7 (21.9)	25 (78.1)	2.953, 0.127	11 (34.4)	21 (65.6)	2.153, 0.341	5 (15.6)	27 (84.4)	1.261, 0.532
Forceps 3	0 (0.0)	4 (100.0)		0 (0.0)	4 (100.0)		0 (0.0)	4 (100.0)	
Duration of lactation									
<7 months	13 (25.5)	38 (74.5)	0.529, 0.467	9 (17.6)	42 (82.4)	**3.957**, **0.047**	7 (13.7)	44 (86.3)	0.519, 0.471
>6months	46 (30.9)	103 (69.1)		48 (32.2)	101 (67.8)		15 (10.1)	134 (89.9)	

*n* = number of pregnant women; *X*^2^ = Pearson's chi-square value; *p* = *p* value; *p* significant at <0.05 (2-tailed). ^∗^Near (place of residence close that they can walk to the health facility), far (need to take a vehicle to health facility). ^∗∗^High income (>GHS1,000.00), middle income (GHS500.00–1,000.00), and low income (<GHS500.00). ^#^Engine vehicle = motorbikes or cars. ^**##**^Exercise level is defined as any form of physical activity (walking, farming, etc.): often (at least five times a week), maybe (at least two times a week). Hypocholesterolemia = serum concentration levels of HDL lower than 1.0 mmol/L; hypercholesterolemia = serum concentration levels of LDL greater than 3.3 mmol/L; hypertriglycerides = serum concentration levels of triglycerides higher than 2.2 mmol/L [23]. The values in bold means those analysis was statistically significant.

**Table 5:** Chi-square analysis of dietary factors associated with various types of dyslipidemias.

Variable	Hypocholesterolemia *n* (%)		*X* ^2^, *p*	Hypercholesterolemia *n* (%)		*X* ^2^, *p*	Hypertriglyceride *n* (%)		*X* ^2^, *p*
	Yes	No		Yes	No		Yes	No	
Frequency of fruit intake									
Daily	0 (0.0)	3 (100.0)		24 (29.6)	57 (70.4)		11 (13.6)	70 (86.4)	
Weekly	28 (28.9)	69 (71.1)	1.630, 0.653	23 (23.7)	74 976.3)	4.496, 0.213	9 (9.3)	88 (90.7)	3.012, 0.390
Monthly	5 (26.3)	14 (73.7)		9 (47.4)	10 (52.6)		1 (5.3)	18 (94.7)	
Quantity of fruit intake									
Small	16 (25.4)	47 (74.6)	2.290, 0.514	21 (33.3)	42 (66.7)		5 (7.9)	58 (92.1)	
Moderate	39 (32.5)	81 (67.5)		34 (28.3)	86 (71.7)	3.892, 0.273	15 (12.5)	105 (87.5)	2.621, 0.454
Large	4 (28.6)	10 (71.4)		1 (7.1)	13 (92.9)		1 (7.1)	13 (92.9)	
Frequency of vegetable intake									
Daily	17 (34.7)	32 (65.3)	7.859, 0.049	15 (30.6)	34 (69.4)	0.685, 0.877	5 (10.2)	44 (89.8)	0.446, 0.931
Weekly	35 (25.5)	102 (74.5)		37 (27.0)	100 (73)		15 (10.9)	122 (89.1)	
Monthly	6 (66.7)	3 (33.3)		3 (33.3)	6 (66.7)		1 (11.1)	8 (88.9)	
Quantity of vegetable intake									
Small	8 (26.7)	22 (73.3)	2.023, 0.568	5 (16.7)	25 (83.3)	2.828, 0.419	1 (3.3)	29 (96.7)	2.814, 0.421
Moderate	37 (28.0)	95 (72.0)		40 (30.3)	92 (69.7)		15 (11.4)	117 (88.6)	
Large	13 (39.4)	20 (60.6)		11 (33.3)	22 (66.7)		5 (15.2)	28 (84.8)	
Frequency of butter intake									
Daily	11 (25.6)	32 (74.4)	4.668, 0.198	8 (18.6)	35 (81.4)	3.068, 0.381	6 (14.0)	37 (86.0)	2.689, 0.442
Weekly	3 (15.0)	17 (85.0)		5 (25.0)	15 (75.0)		1 (5.0)	19 (95.0)	
Monthly	45 (33.6)	89 (66.4)		43 (32.1)	91 (67.9)		14 (10.4)	120 (89.6)	
Quantity of butter intake									
Small	23 (22.3)	80 (77.7)	6.473, 0.091	30 (29.1)	73 (70.9)	0.244, 0.970	10 (9.7)	93 (90.3)	1.771, 0.621
Moderate	29 (39.7)	44 (60.3)		21 (28.8)	52 (71.2)		10 (13.7)	63 (86.3)	
Large	6 (31.6)	13 (68.4)		14 (73.7)	14 (73.7)		1 (5.3)	18 (94.7)	
Frequency of egg intake									
Daily	9 (26.5)	25 (73.5)	2.326, 0.508	8 (23.5)	26 (76.5)	1.383, 0.710	3 (8.8)	31 (91.2)	2.257, 0.521
Weekly	29 (33.9)	57 (66.3)		28 (32.6)	58 (67.4)		8 (9.3)	78 (90.7)	
Monthly	21 (27.3)	56 (72.7)		20 (26.0)	57 (74.0)		10 (13.0)	67 (87.0)	
Quantity of egg intake									
Small	25 (26.9)	68 (73.1)	2.459, 0.483	28 (30.1)	65 (69.9)	0.644, 0.886	7 (7.5)	86 (92.5)	2.527, 0.470
Moderate	25 (29.8)	59 (70.2)		24 (28.6)	60 (71.4)		11 (13.1)	73 (86.9)	
Large	8 (44.4)	10 (55.6)		4 (22.2)	14 (77.8)		3 (16.7)	15 (83.5)	
Frequency of milk intake									
Daily	26 (28.0)	67 (72.0)	1.776, 0.620	29 (31.2)	64 (68.8)	1.612, 0.657	9 (9.7)	84 (90.3)	1.726, 0.631
Weekly	12 (34.3)	23 (65.7)		7 (20.0)	28 (80.0)		4 (11.4)	31 (88.6)	
Monthly	21 (30.4)	48 (69.6)		20 (29.0)	49 (71.0)		8 (11.6)	61 (88.4)	
Quantity of milk intake									
Small	13 (25.0)	39 (75.0)	2.079, 0.556	12 (23.1)	40 (76.9)	1.202, 0.753	6 (11.5)	46 (88.5)	1.387, 0.709
Moderate	30 (28.6)	75 (71.4)		33 (31.4)	72 (68.6)		13 (12.4)	92 (87.6)	
Large	14 (38.9)	22 (61.1)		10 (27.8)	26 (72.2)		2 (5.6)	34 (94.4)	
Frequency of meat intake									
Daily	34 (30.1)	79 (69.9)	1.754, 0.625	32 (28.3)	81 (71.7)	1.283, 0.733	12 (10.6)	101 (89.4)	3.538, 0.316
Weekly	20 (31.7)	43 (68.3)		20 (31.7)	43 (68.3)		5 (7.9)	58 (92.1)	
Monthly	5 (23.8)	16 (76.2)		4 (19.0)	17 (81.0)		4 (19.0)	17 (81.0)	
Quantity of meat intake									
Small	17 (37.8)	28 (62.2)	5.735, 0.125	13 (28.9)	32 (71.1)	6.025, 0.091	4 (8.9)	41 (91.1)	3.098, 0.377
Moderate	23 (22.1)	81 (77.9)		36 (34.6)	68 (65.4)		9 (8.7)	95 (91.3)	
Large	17 (37.8)	28 (62.2)		38 (84.4)	38 (84.4)		8 (17.8)	37 (82.2)	
Frequency of legume intake									
Daily	8 (20.5)	31 (79.5)	2.900, 0.407	10 (25.6)	29 (74.4)	4.263, 0.234	6 (15.4)	33 (84.6)	4.426, 0.219
Weekly	32 (32.7)	66 (67.3)		24 (24.5)	74 (75.5)		10 (10.2)	88 (89.8)	
Monthly	19 (31.1)	42 (68.9)		23 (37.7)	38 (62.3)		5 (8.2)	56 (91.8)	
Quantity of legume intake									
Small	13 (31.0)	29 (69.0)	2.323, 0.508	13 (31.0)	29 (69.0)	0.773, 0.856	4 (9.5)	38 (90.5)	0.397, 0.941
Moderate	31 (33.3)	62 (66.7)		25 (26.9)	68 (73.1)		11 (11.8)	82 (88.2)	
Large	13 (22.0)	46 (78.0)		18 (30.5)	41 (69.5)		6 (10.2)	53 (89.8)	
Frequency of grain intake									
Daily	37 (28.5)	93 (71.5)	2.889, 0.409	38 (29.2)	92 (70.8)	1.248, 0.741	15 (11.5)	115 (88.5)	4.196, 0.241
Weekly	21 (35.0)	39 (65.0)		16 (26.7)	44 (73.3)		6 (10.0)	54 (90.0)	
Monthly	1 (12.5)	7 (87.5)		3 (37.5)	5 (62.5)		0 (0.0)	8 (100.0)	
Quantity of grain intake									
Small	8 (25.0)	24 (75.0)	0.495, 0.920	8 (25.0)	24 (75.0)	0.729, 0.866	3 (9.4)	29 (90.6)	3.271, 0.352
Moderate	27 (30.3)	62 (69.7)		28 (31.5)	61 (68.5)		7 (7.9)	82 (92.1)	
Large	22 (31.0)	49 (69.0)		19 (26.8)	52 (73.2)		10 (14.1)	61 (85.9)	
Frequency of fish intake									
Daily	41 (29.9)	96 (70.1)	0.939, 0.816	36 (26.3)	101 (73.7)	2.223, 0.527	18 (13.1)	119 (86.9)	6.280, 0.099
Weekly	16 (30.2)	37 (69.8)		18 (34.0)	35 (66.0)		3 (5.7)	50 (94.3)	
Monthly	2 (25.0)	6 (75.0)		3 (37.5)	5 (62.5)		0 (0.0)	8 (100.0)	
Quantity of fish intake									
Small	12 (27.3)	32 (72.7)	2.519, 0.472	16 (36.4)	28 (63.6)	1.829, 0.609	6 (13.6)	38 (86.4)	
Moderate	38 (32.5)	79 (67.5)		30 (25.6)	87 (74.4)		14 (12.0)	103 (88.0)	3.595, 0.309
Large	9 (25.7)	26 (74.3)		10 (28.6)	25 (71.4)		1 (2.9)	34 (97.1)	

*n* = number of pregnant women; *X*^2^ = Pearson's chi-square value; *p* = significance at <0.05 (2-tailed).

**Table 6:** Result of multinomial logistic regression analysis.

Dyslipidemia type	Parameter		*B*	Standard error	Exp (B)/AOR	95% CI AOR	*p*
Hypocholesterolemia	Age		-0.038	0.029	0.963	0.910-1.019	**<0.001**
	Religion	Muslim Christianity	0.030 0^b^	0.495	1.030	0.391-2.716	0.952
	Educational status	Educated Noneducated	-1.008 0^b^	0.490	0.365	0.140-0.954	**0.040**
	Occupation	Employed Unemployed	-0.313 0^b^	0.452	0.731	0.301-1.773	0.488
	Residence	Urban Rural	-0.060 0^b^	0.517	0.942	0.342-2.595	0.908
	Income	Low Middle High	0.610 0.602 0^b^	0.807 0.744	1.841 1.825	0.378-8.955 0.425-7.840	0.450 0.418
	Exercise level	None Often Maybe	-0.253 -0.335 0^b^	0.486 0.447	0.777 0.715	0.299-2.015 0.298-1.717	0.603 0.453
	Alcohol intake	No Yes	0.450 0^b^	0.823	1.568	0.312-7.875	0.585
	Type of breastfeeding	Nonexclusive Exclusive	-0.027 0^b^	0.403	0.974	0.442-2.145	0.947
	Duration of lactation	>6 months <6 months	-0.282 0^b^	0.425	0.754	0.328-1.733	0.506
	Delivery outcome	Twin Singleton	0.027 0^b^	0.628	1.027	0.300-3.518	0.966
	Parity	Uniparous Multiparous	0.015 0^b^	0.388	1.015	0.474-2.174	0.969

Hypercholesterolemia	Age		-0.50	0.031	0.951	0.896-1.010	0.103
	Religion	Muslim Christianity	-0.980 0^b^	0.488	0.375	0.144-0.978	**0.045**
	Educational status	Educated Noneducated	-0.769 0^b^	0.554	0.464	0.156-1.374	0.165
	Occupation	Employed Unemployed	-0.637 0^b^	0.486	0.529	0.204-1.374	0.529
	Residence	Urban Rural	0.363 0^b^	0.605	1.438	0.440-4.705	0.548
	Income	Low Middle High	-2.154 -1.813 0^b^	0.760 0.665	0.116 0.163	0.026-0.514 0.044-0.600	**0.005** **0.006**
	Exercise level	None Often Maybe	-0.537 -0.265 0^b^	0.508 0.468	0.585 0.768	0.216-1.583 0.307-1.920	0.585 0.768
	Alcohol intake	No Yes	1.842 0^b^	0.888	6.312	1.108-35.949	**0.038**
	Type of breastfeeding	Nonexclusive Exclusive	-0.242 0^b^	0.425	0.785	0.342-1.805	0.569
	Duration of lactation	>6 months <6 months	-0.880 0^b^	0.467	0.415	0.166-1.037	0.060
	Delivery outcome	Twin Singleton	-0.743 0^b^	0.739	0.476	0.112-2.023	0.314
	Parity	Uniparous Multiparous	0.014 0^b^	0.400	0.986	0.450-2.160	0.973

Hypertriglycerides	Age		0.004	0.044	1.004	0.922-1.093	0.930
	Religion	Muslim Christianity	0.400 0^b^	0.763	1.492	0.335-6.657	0.600
	Educational status	Educated Noneducated	0.393 0^b^	0.798	0.622	0.310-7.075	0.622
	Occupation	Employed Unemployed	1.003 0^b^	0.714	1.482	0.673-11.055	0.160
	Residence	Urban Rural	0.280 0^b^	0.796	1.324	0.278-6.296	0.725
	Income	Low Middle High	0.906 -0.906 0^b^	1.093 1.019	2.475 0.403	0.291-21.079 0.055-2.968	0.407 0.372
	Exercise level	None Often Maybe	0.206 -0.562 0^b^	0.722 0.707	1.228 0.570	0.298-5.057 0.143-2.2279	0.776 0.427
	Alcohol intake	No Yes					
	Type of breastfeeding	Nonexclusive Exclusive	0.703 0^b^	0.591	2.019	0.634-5.428	0.234
	Duration of lactation	>6 months <6 months	0.995 0^b^	0.582	2.704	0.865-8.458	0.087
	Delivery outcome	Twin Singleton	-0.064 0^b^	1.167	0.938	0.095-9.237	0.956
	Parity	Uniparous Multiparous	0.533 0^b^	0.593	1.704	0.533-5.445	0.369

*p* is analyzed by multinomial logistic regression analyses and considered significant at <0.05 (2-tailed). The reference category is normal. ^b^Parameter considered redundant and set to zero. *B*: regression coefficient; ExpB: exponentiation of B, which is the same as AOR; AOR: adjusted odds ratio; 95% CI: 95% confidence interval. The values in bold means the analysis were statistically significant.

## Data Availability

The data to the study is available upon reasonable request.
